# Supplementary material for: Differences in the expression profiles of lncRNAs and mRNAs in partially injured anterior cruciate ligament and medial collateral ligament of rabbits
Source: PeerJ. 2022 Jan 12;10:e12781. doi: 10.7717/peerj.12781 (PMC8760859; doi:10.7717/peerj.12781)
Supplement: Supplemental Information 1 — F: Forward; lncRNA: Long noncoding RNA; R: reverse. [file peerj-10-12781-s001.docx]

**Supplementary Table 1 Primers used for quantitative real-time polymerase chain reaction**

| **Gene** | **Type** | **Primer (5′–3′)** |  |
| --- | --- | --- | --- |
| MSTRG.86040.7 | lncRNA | F:TGGAGAGGCAAAGGCAGAGAGG  R:GGAGTGAACCAACAGACGGAAGAC | |
| MSTRG.24371.1 | lncRNA | F:TGCCTTTGACCTTCCGTGCTATG  R:AGCCCAGAGCGACTGACTTCC | |
| MSTRG.58645.12 | lncRNA | F:CAGAGAAGTGGACAGTGCGGTTC  R:AGGACAGTAGCAGAGCAAGAGGAG | |
| MSTRG.27151.1 | lncRNA | F:TGGCACAAGTAGAAGGGAGCTAGG  R:GTTGACAAGGAGGAGGAGGAGGAG | |
| MSTRG.75325.14 | lncRNA | F:GCAGCGAACTAATGGCAAAGGAAG  R:AGGCAGAGTGGACAGTGAGTGAG | |
| MSTRG.87246.18 | lncRNA | F:ATGGCAGAGACGCAAGCACTTC  R:GTCCTGACGCTCCACTTCCAATAC | |
| MSTRG.69384.5 | lncRNA | F:CTGTGAATAGCCACTGCACTCCAG  R:AGGTCACCATATTGATGCCGAACTTAG | |
| MSTRG.86040.9 | lncRNA | F:GGAGTTTCAGAGATGGAGAGGCAAAG  R:GGAGTGAACCAACAGACGGAAGAC | |
| MSTRG.25442.2 | lncRNA | F:TAAAGGACAGGCATTTGGCAGAGTG  R:CAGGAGCAGAGCATCATGGACTTG | |
| MSTRG.90026.2 | lncRNA | F:TGGAGTCTGGTTGGGAGCCTTATC  R:TGGAGTCTGGTTGGGAGCCTTATC | |
| MSTRG.27082.4 | lncRNA | F:TCATCCACGGATTAGTTCTTGCTTCAG  R:ATGTCTCAATCAGTCTCGCCGAATG | |
| MSTRG.102609.16 | lncRNA | F:TTCCCTTCGCAGACCTTCATTTCAC  R:ACAAGAACAGAACGCAGAGCCAAG | |
| MSTRG.68818.7 | lncRNA | F:TCTCACTGTCCACTCTGCCTGTC  R:TCTCACTGTCCACTCTGCCTGTC | |
| MSTRG.22927.1 | lncRNA | F:AACCGATGCCCTCTTTGATCTGAAC  R:TTCCTTGCTCCACCTGCTGTTTG | |
| MSTRG.25430.16 | lncRNA | F:ATCTCTCTGCCTCTCCTTTCTCTCTG  R:CTTAACCTCTGTGTTCTTTCCCTCCTC | |
| MSTRG.90336.3 | lncRNA | F:TCTCACTGTCCACTCTGCCTGTC  R:CTGCTGTGCCAGTATGTTACCTCTC | |
| MSTRG.45608.5 | lncRNA | F:GTGCCCACATCTCATACCCTAATGC  R:GAGCCATCACTTACTGCCTTCCTTG | |
| MSTRG.41179.7 | lncRNA | F:ACTGGCTGCTTCCTCCTCATATCC  R:GTGTCCTGTATGTACTGCGTCCAAG | |
| MSTRG.42937.5 | lncRNA | F:CTTGTATCGTGGCAAAGTGGGTTAAAC  R:TAGGCACATCAGCAGAGGCAGAG | |
| MSTRG.32775.12 | lncRNA | F:GTGAAGGACATAGCGAGCCAGATG  R:AGAGAAGCAGCACAAGCCTCATTC | |
| LPL | mRNA | F:TGGGCTGTGTAGATAGTGGGAAGG  R:TTACGAGACCAACGCTGCTGAAAG | |
| COL6A6 | mRNA | F:CTGGCATTGTGGTAGACTGAGTAAGG  R:AGCAGGGAGTTGGATTGTAATTGGAG | |
| PPP2R2B | mRNA | F:ACAACACAATCCGCAGACTTAGACC  R:TTCAGGCACCATTTCAAGACTCAGAC | |
| LIF | mRNA | F:TTCCTGTTTGCACCATTTCCTCTCC  R:TTGCTCCTCTACTGTCCCTTGTCC | |
| IBSP | mRNA | F:TGCGAGGAATTTATGTGGAACTGAGG  R:GTTAGGTGATGAGCATGGATGGAGAC | |
| MMP9 | mRNA | F:AGTGTCTGCTCTCATCTCCTCAGTAC  R:AGTGTCTGCTCTCATCTCCTCAGTAC | |
| CACNA1D | mRNA | F:CGTCTCGTAACATCAGCAGGTCTAAG  R:TAAGCAATCAGCACCAGGCACTTG | |
| HHIP | mRNA | F:CCTTCCTTGTGTTGGCTTCATTCTTG  R:AGGGCTAAGTCTGGAGTCTGAGTTC | |
| WNT10B | mRNA | F:AGGGCTAAGTCTGGAGTCTGAGTTC  R:CAGGACTCAAGAAAGCCAGGGAAC | |
| PIK3CD | mRNA | F:TGATCTCCTGTCTGCCTTCACCTC  R:AAGTCCACCGCCACATTCTGATTC | |
| RAC2 | mRNA | F:CTGCTCACTTCTGCTTTGGGTCTG  R:CAAGGTAACTCCGAATGGCTGGAAG | |
| COL9A2 | mRNA | F:TCTCGTTATAGCGTCAAATAGCCAGTC  R:GGTAAAGAAACACAGCAGCCAAAGG | |
| SMOC2 | mRNA | F:CTGGTACTTCAAGCTCCTGGACAAG  R:CCACGAACTTCTTCACGCATTTCTTC | |
| FGFR2 | mRNA | F:CTATGCTTGTACTGCTGCTAGGACTG  R:TCTCTTGCTGTTACTGTTCTCACTGAC | |
| POSTN | mRNA | F:TCCTAATTCCTGACTCTGCCAAACAAG  R:AGGTGCCAGCAAAGTGTATTCTCC | |
| CILP | mRNA | F:CAGACAGACAGCAATGGGAGGTTC  R:GTGAGCACAATGGGAGCAAACTTG | |
| SEMA3E | mRNA | F:CCTGCCCACAAGAAACCCATACTG  R:GTCCTCCGCTTCTACCCGATCC | |
| ZNF385B | mRNA | F:GCCAAGAAGGTCAAAGCACTAGAGG  R:TGTTGCCTGTGATTGGAGTGATGG | |
| MFAP4 | mRNA | F:AGGAAGACGGCTACACTCTCTACG  R:CGGTCGAAGGTGGAGAATTTCTGG | |
| TBX3 | mRNA | F:CAACATCTCGGACAAGCATGGATTTAC  R:TCATTGGCTCTTACGATGTGGAACC | |
| FBLN5 | mRNA | F:ACCGCTCTCTGCTCCCAACTAC  R:GCACACTCGTCCACATCCACAC | |
| ITGB8 | mRNA | F:AATGCCTTCACCCTCACAATCTGTC  R:CACTCTGATGCCTGCTCCACATAAG | |
| HHIP | mRNA | F:CCTTCCTTGTGTTGGCTTCATTCTTG  R:AGGGCTAAGTCTGGAGTCTGAGTTC | |
| β-actin | Maker | F:CAGATGTGGATCAGCAAGCAGGAG  R:TAGGTTTCGTCGAGAGAGGGTGTC | |

F: Forward; lncRNA: Long noncoding RNA; R: reverse.
