# Supplementary figures and images for: Differences in the expression profiles of lncRNAs and mRNAs in partially injured anterior cruciate ligament and medial collateral ligament of rabbits
Source: PeerJ. 2022 Jan 12;10:e12781. doi: 10.7717/peerj.12781 (PMC8760859; doi:10.7717/peerj.12781)

**Supplementary Table 2. Sample sequencing data evaluation statistics**

**
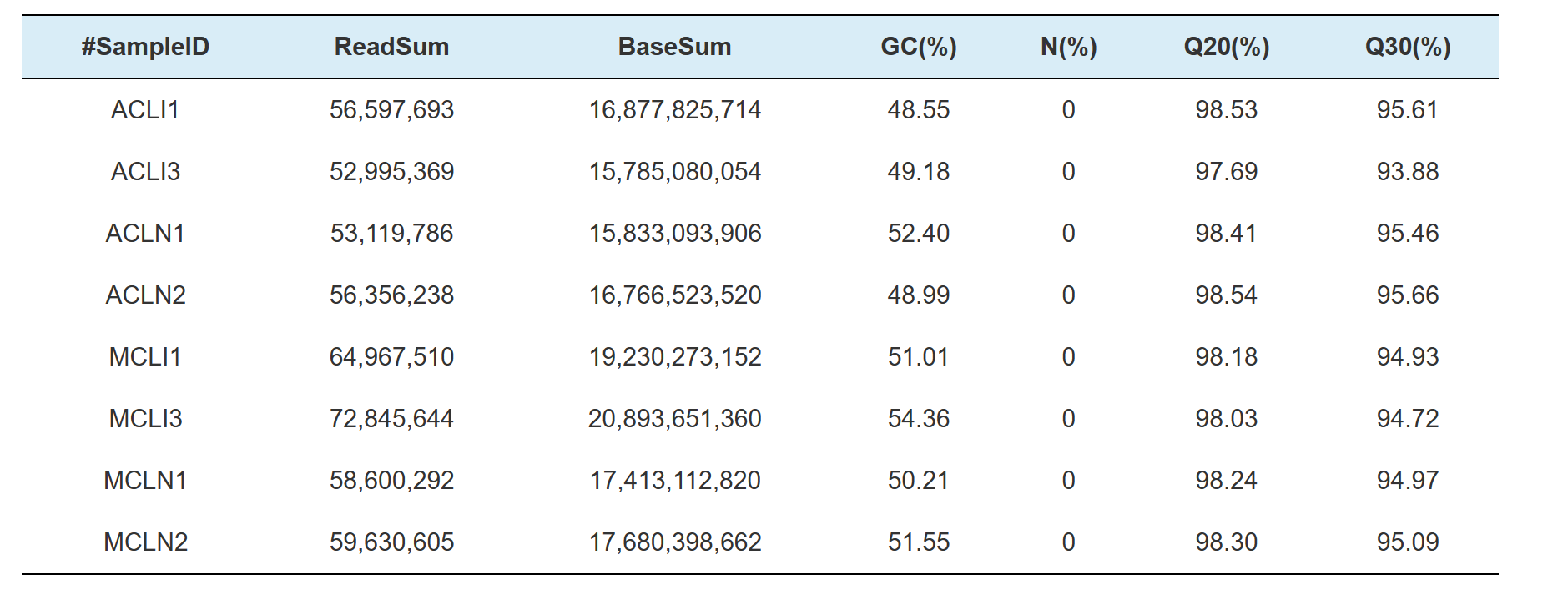
**

Supplement: Supplemental Information 2 — SampleID: sample name. ReadSum: the total number of pair-end Reads in Clean Data. BaseSum: the total number of nucleobase in Clean Data. GC(%): Clean Data GC content, i.e., the percentage of both G and C bases in Clean Data out of the total nucleobase. N(%): the percentage of unresolved nucleobase in Clean Data to the total nucleobase. Q30 (%): the percentage of nucleobase with Clean Data mass value greater than or equal to Q30. [file peerj-10-12781-s002.docx]

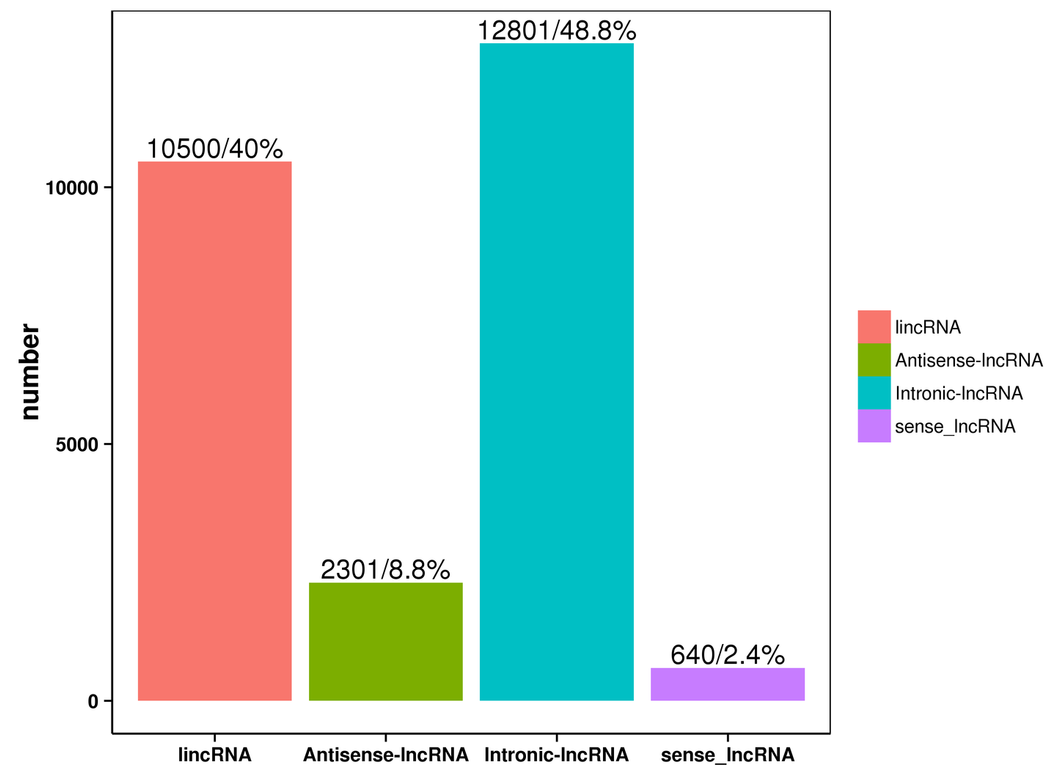

Supplement: Supplemental Information 3 [file peerj-10-12781-s003.pdf]

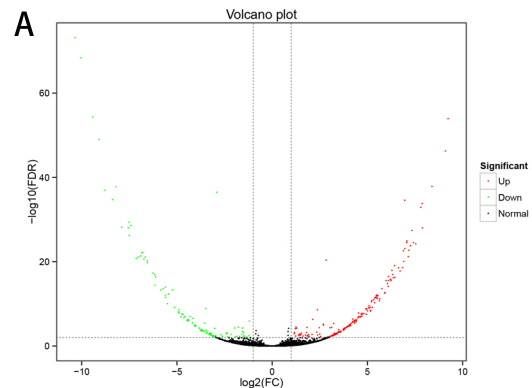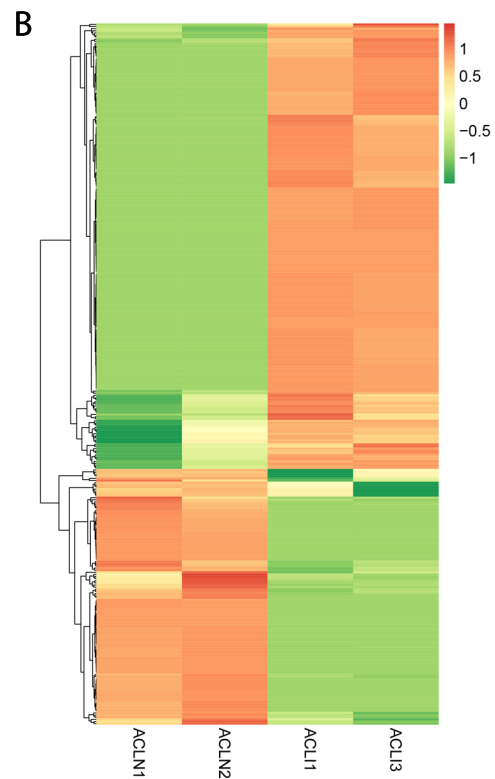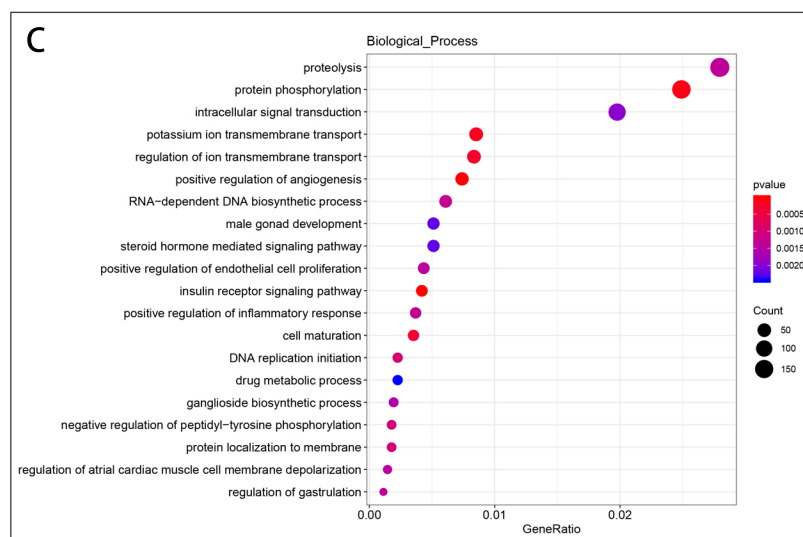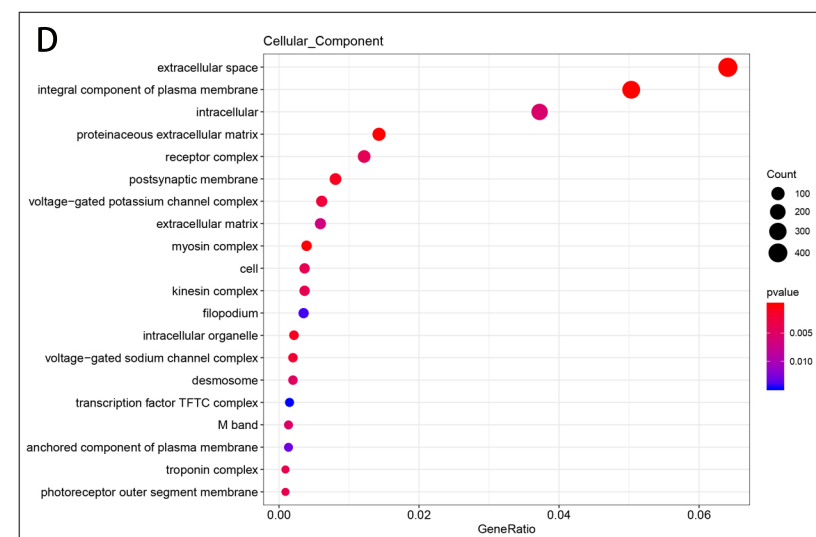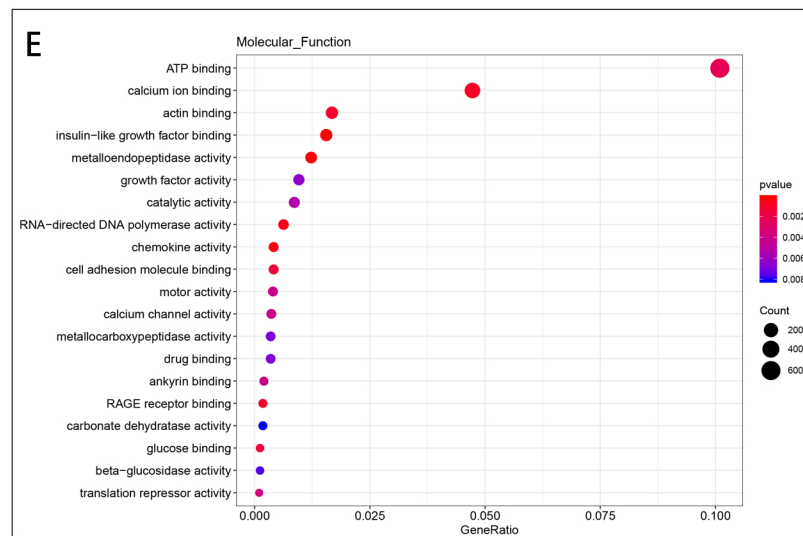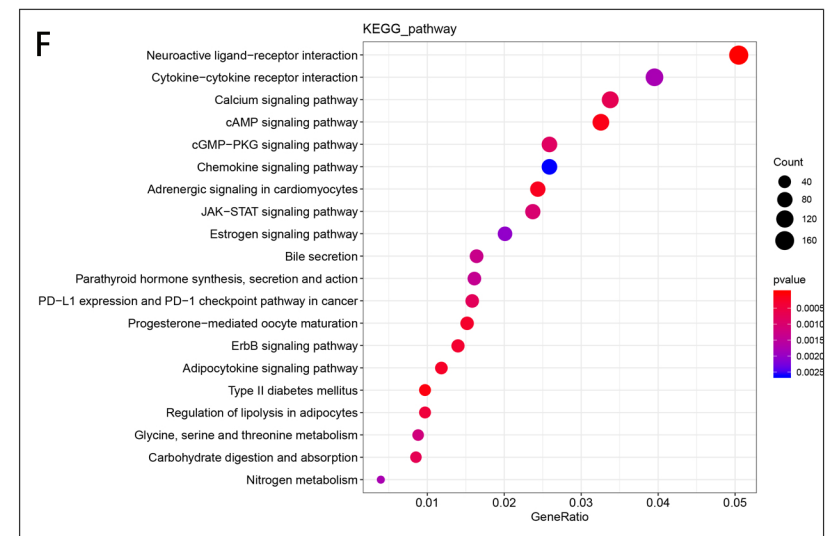

Supplement: Supplemental Information 4 — (A) Volcano plot of the differentially expressed lncRNAs. The red and green dots represent statistically significantly up-regulated and down-regulated lncRNAs. (B) Hierarchical clustering shows a difference in lncRNA expression profile between the two groups and homogeneity within groups. (C–E) Top 20 highest enriched GO terms for target genes of DELs. (F) Top 20 highest enriched KEGG pathways for target genes of DELs. [file peerj-10-12781-s004.pdf]

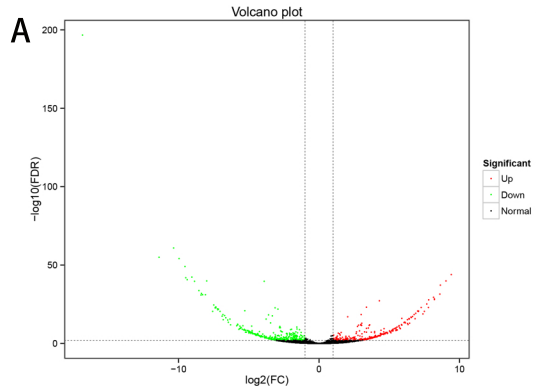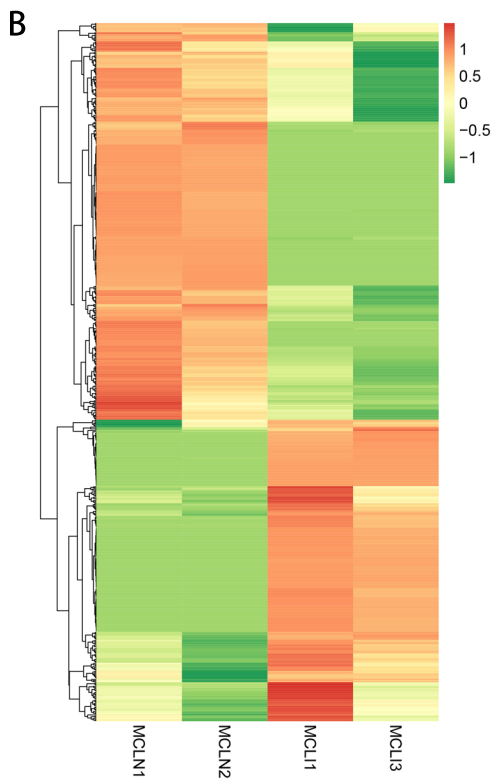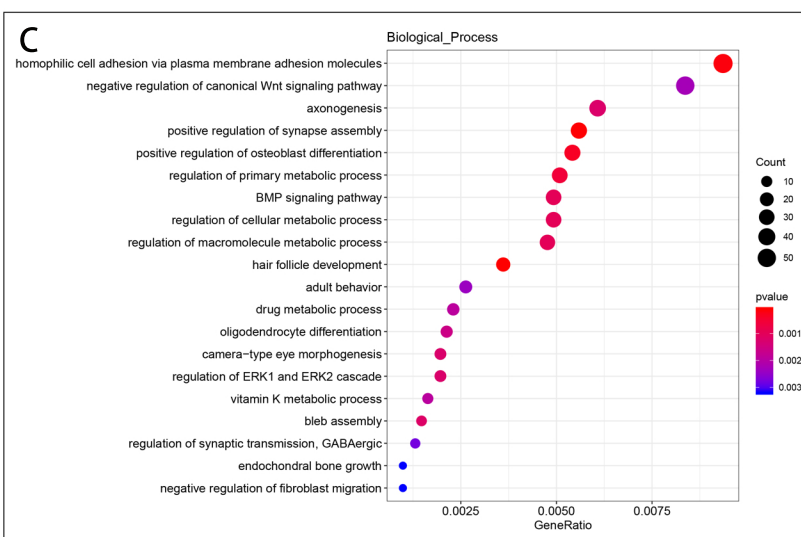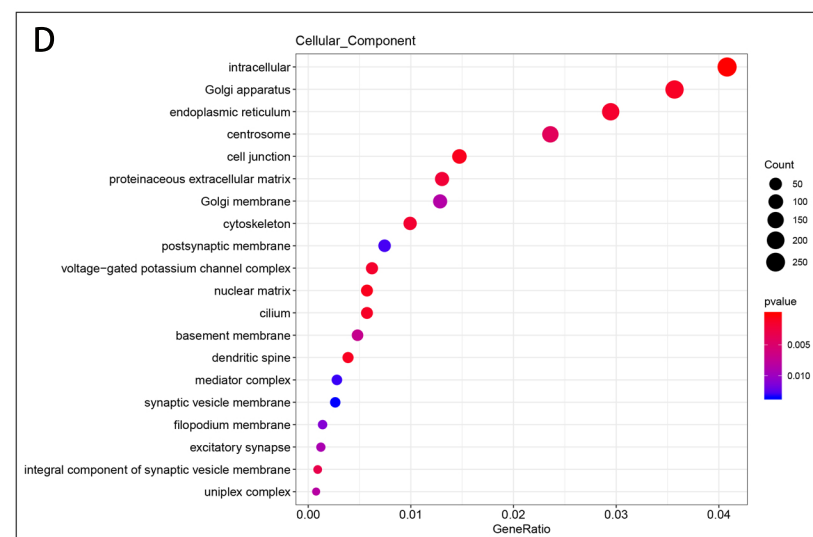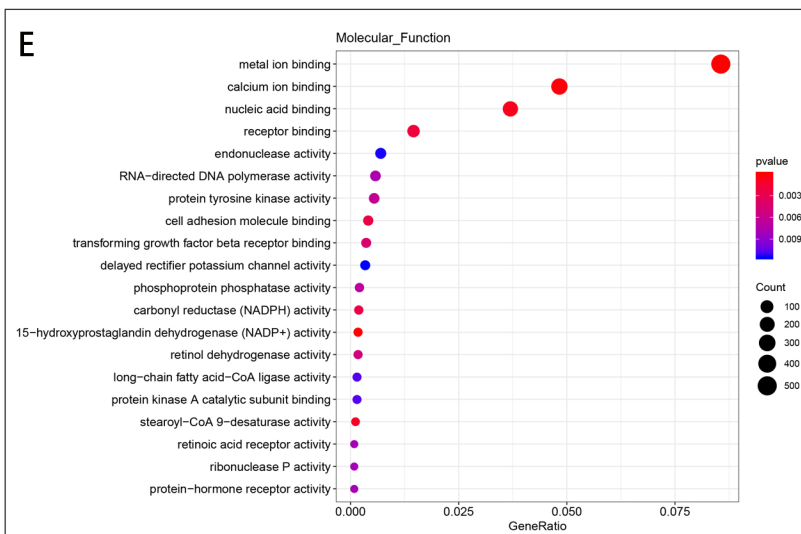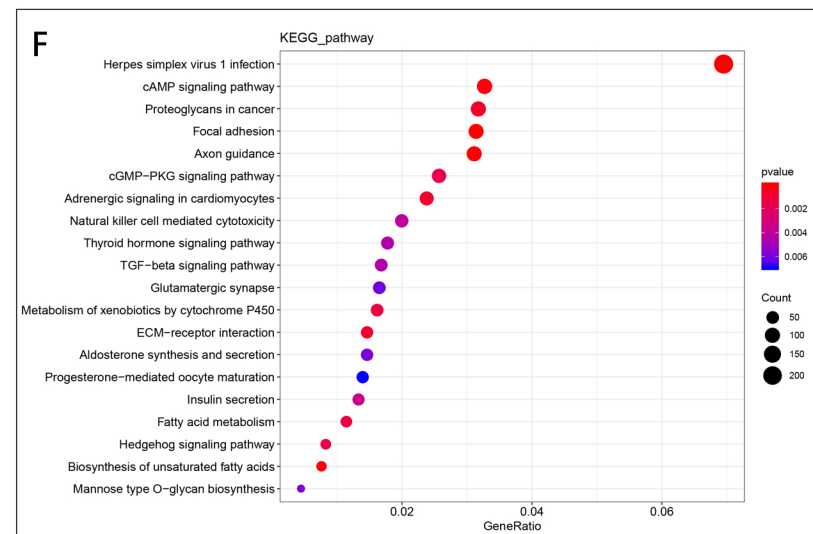

Supplement: Supplemental Information 5 — (A) Volcano plot of the differentially expressed lncRNAs. The red and green dots represent statistically significantly up-regulated and down-regulated lncRNAs. (B) Hierarchical clustering shows a difference in lncRNA expression profile between the two groups and homogeneity within groups. (C–E) Top 20 highest enriched GO terms for target genes of DELs. (F) Top 20 highest enriched KEGG pathways for target genes of DELs. [file peerj-10-12781-s005.pdf]

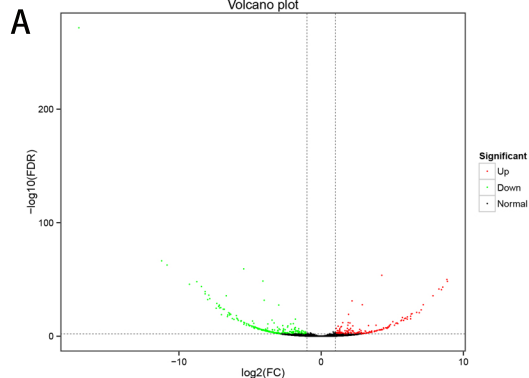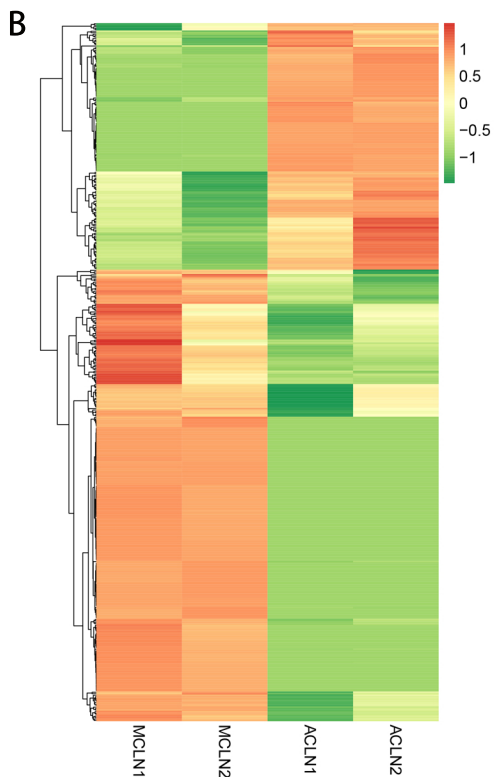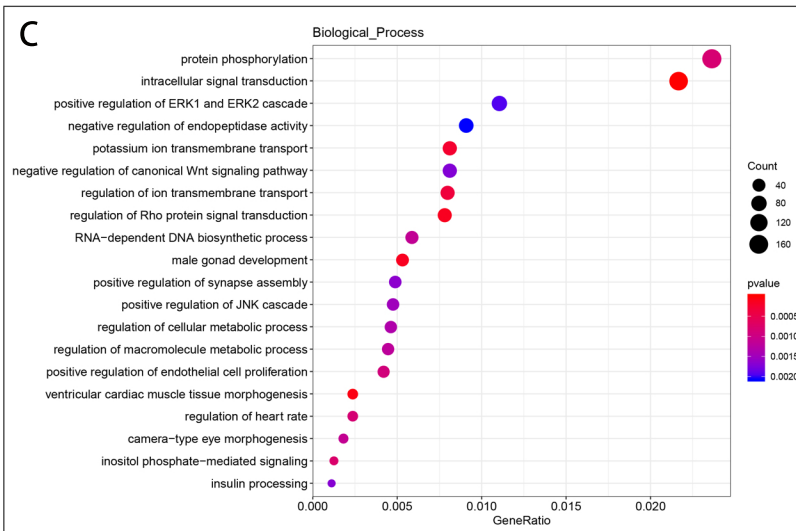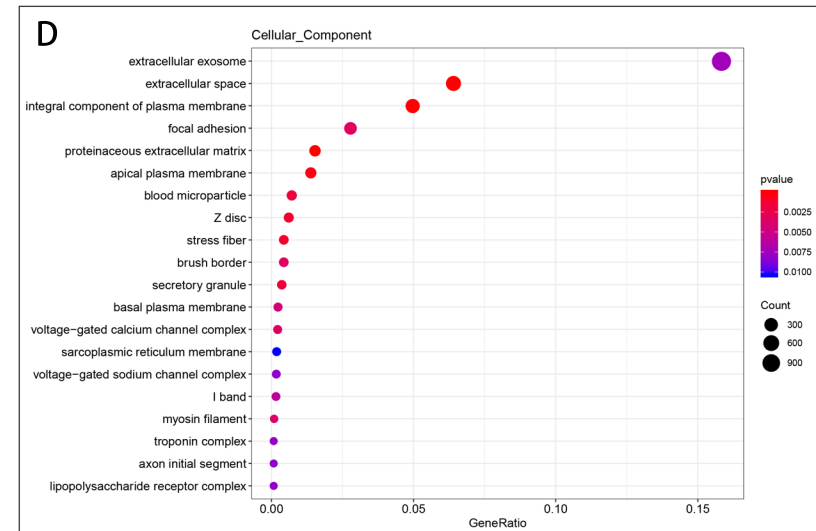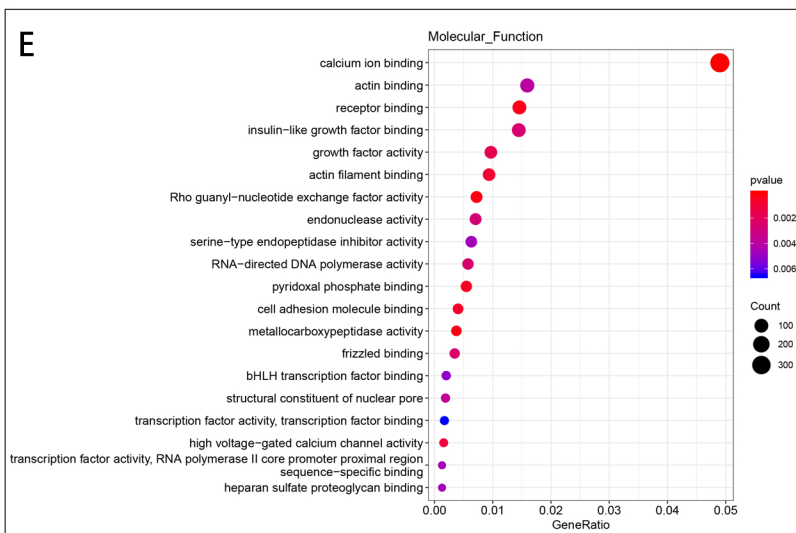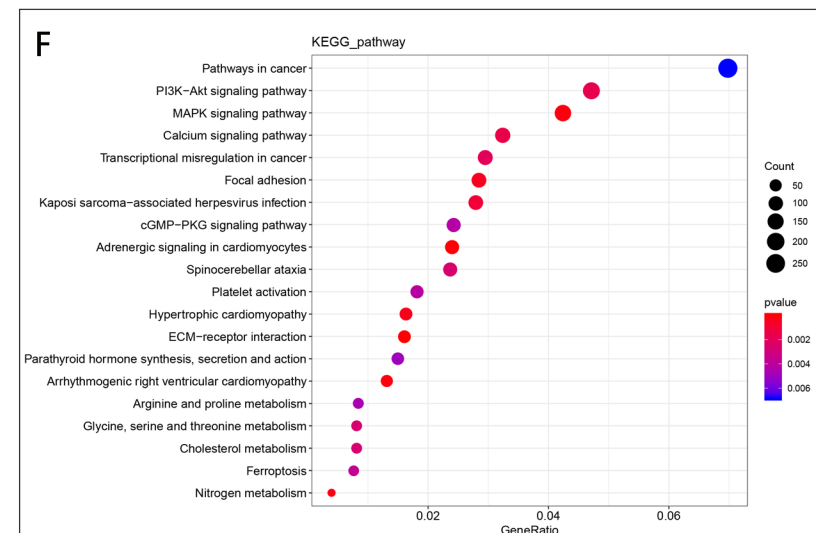

Supplement: Supplemental Information 6 — (A) Volcano plot of the differentially expressed lncRNAs. The red and green dots represent statistically significantly up-regulated and down-regulated lncRNAs. (B) Hierarchical clustering shows a difference in lncRNA expression profile between the two groups and homogeneity within groups. (C–E) Top 20 highest enriched GO terms for target genes of DELs. (F) Top 20 highest enriched KEGG pathways for target genes of DELs. [file peerj-10-12781-s006.pdf]

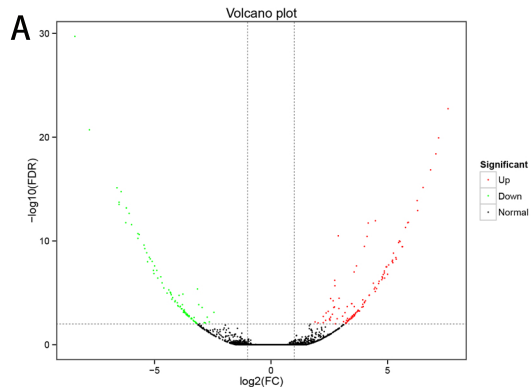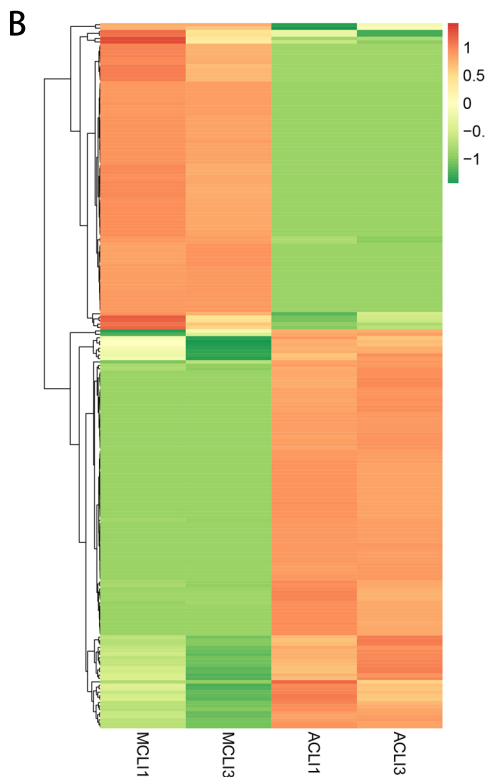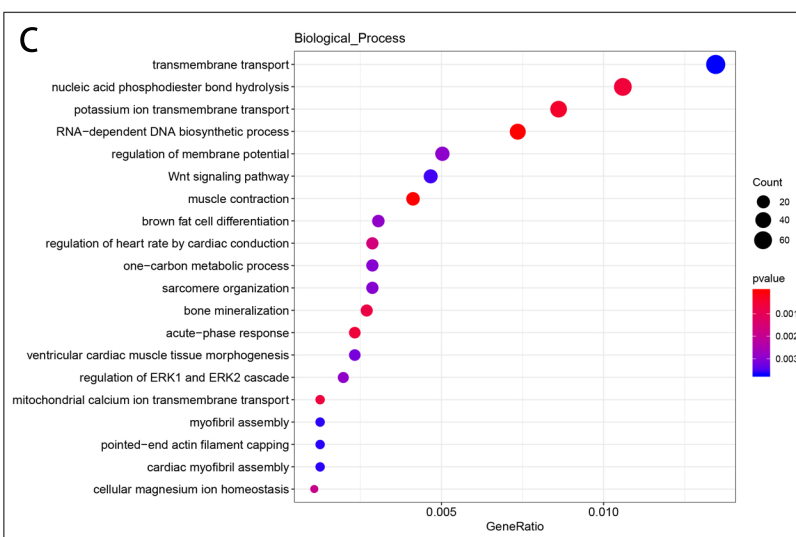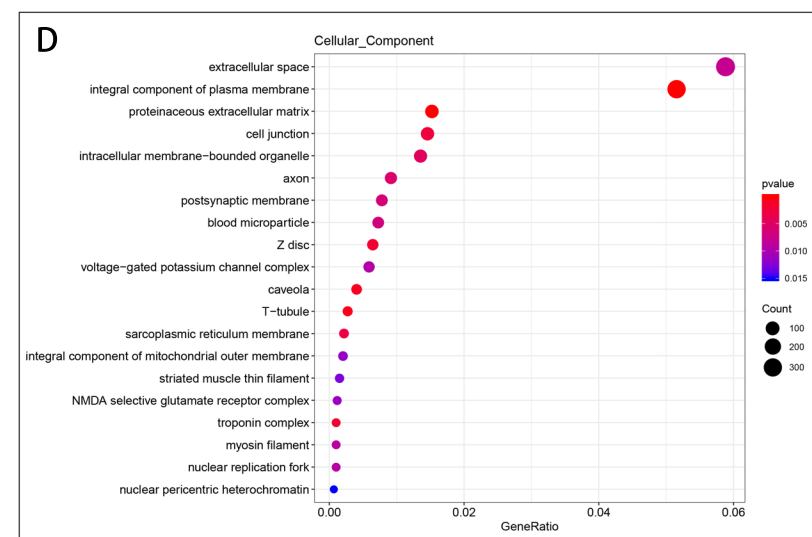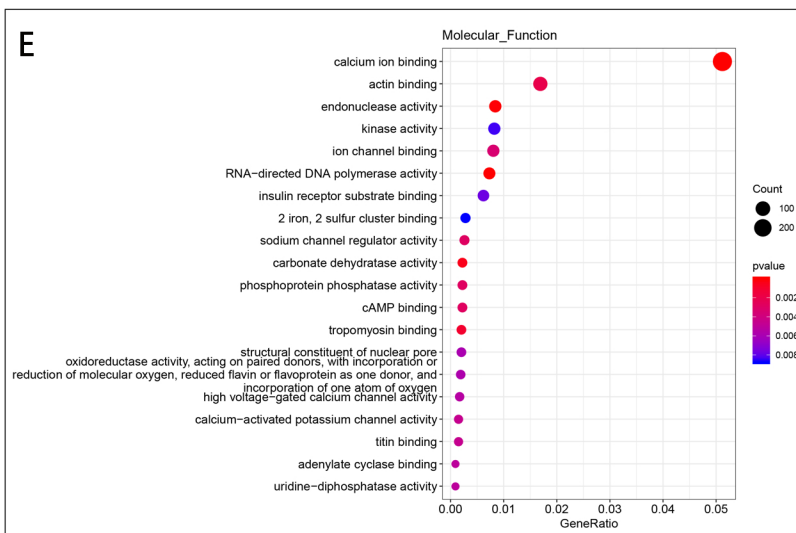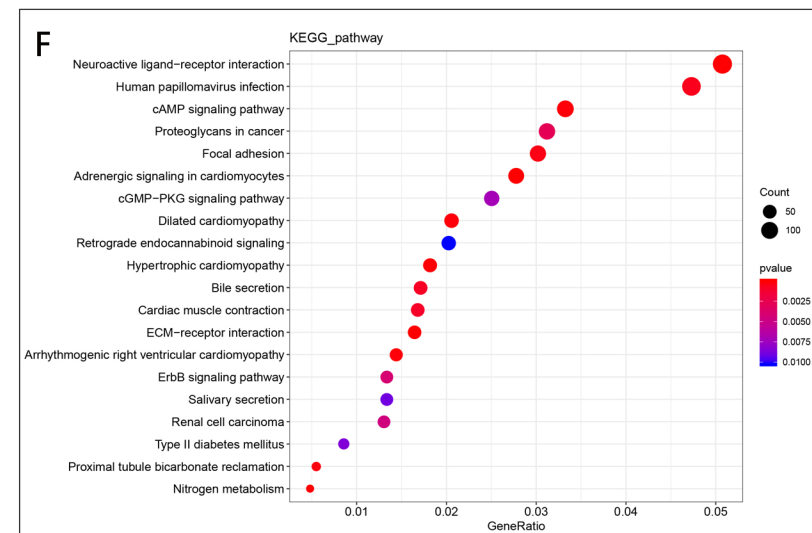

Supplement: Supplemental Information 7 — (A) Volcano plot of the differentially expressed lncRNAs. The red and green dots represent statistically significantly up-regulated and down-regulated lncRNAs. (B) Hierarchical clustering shows a difference in lncRNA expression profile between the two groups and homogeneity within groups. (C–E) Top 20 highest enriched GO terms for target genes of DELs. (F) Top 20 highest enriched KEGG pathways for target genes of DELs. [file peerj-10-12781-s007.pdf]
